# GDMT for heart failure and the clinician's conundrum

**DOI:** 10.1002/clc.23268

**Published:** 2019-09-16

**Authors:** Padmaraj Samarendra

**Affiliations:** ^1^ VA Medical Center Pittsburgh Pennsylvania; ^2^ University of Pittsburgh, School of Medicine Pittsburgh Pennsylvania

## Abstract

Therapeutic advances in management of CHF have decreased mortality and have impacted progression in patients with mild to moderate heart failure. Aggressive campaigns by cardiology societies aimed at increasing implementation of these measures in routine practices have almost generalized the treatment of heart failure irrespective of individual variations of clinical status of patients and stages of heart failure. This explains why morbidity compression and quality of life improvement have not been realized fully particularly in patients with advanced disease. To examine whether GDMT for CHF is backed by unambiguous evidence of clinical efficacy for its global implementation in every patient at all stages of the syndrome. ACC/AHA, ESC Guidelines for CHF, and their updates were reviewed. Clinical trial cited in the guideline documents and other pertaining published literatures were analyzed.

**Findings:**

Many of the recommended GDMT for CHF lack unequivocal evidence of clinical efficacy in patients with diverge etiology of heart failure and concomitant comorbid conditions Some of the recommendations which are useful in early stages, lack evidence of efficacy in more advanced stages of heart failure. Application of results of research trials in patients beyond their inclusion and exclusion criteria, appears mere extrapolation, Clinicians are faced with the conundrum of implementing the recommendations without indubitable evidence of their efficacy in every patient of their practice.

**Conclusion:**

A reappraisal of Guidelines is needed to address outstanding questions pertaining to the efficacy of recommendations and plug the knowledge gaps without assumption and extrapolation of results of RCTs beyond their inclusion and exclusion criteria.

**ABBREVIATIONS**
ACE‐I: angiotensin converting enzyme inhibitor
ARB: angiotensin receptor blocker
HF: heart failure
HFpEF: heart failure preserved ejection fraction
HFrEF: heart failure reduced ejection fraction
GDMT: Guideline‐Directed Medical Therapy
GWTG: Get with The Guideline
NYHA: New York Heart Association Functional Class
PA: pulmonary artery
PCWP: pulmonary capillary wedge pressure
SBP: systolic blood pressure

Therapeutic advances made in the management of heart failure (HF) in the last three decades are now the cornerstone of guidelines developed by cardiology societies.^1^, ^2^ Undesirably Guideline‐Directed Medical Therapy (GDMT) has generalized the management of heart failure despite differences in the clinical manifestation of the syndrome in different or even the same patients.

The progressive nature of heart failure with changing pathophysiology impacted by comorbidities and even therapies used, makes treatment that was effective at one stage problematic with disease progression to more advanced stage.

Furthermore, clinical efficacy of GDMT components as shown in clinical trials with restrictive inclusion and exclusion criteria, is often not replicated in routine practice. For example, the benefit and harms from Aldosterone antagonists in the real world are significantly different to the reported results of the RALES trial.^3^ According to one report, only 20 patients are needed to be treated in routine practice for one case of life‐threatening hyperkalemia and harm to occur.^4^

However clinicians are faced with the conundrum of implementing the recommendations without indisputable evidence of their efficacy in patients, coerced by the fact that the level of application of GDMT is used as a Performance Measure Tool.

In‐depth analysis of the guidelines shows gaps in the evidence supporting the recommendations as described here.

## 1 CARDIOVERTER DEFIBRILLATORS FOR PRIMARY PREVENTION OF SUDDEN DEATH

Both ACC/AHA (2013/2017 update) and ESC 2016 guidelines agree regarding use of Defibrillators, which are arguably the most controversial recommendation and have, limited evidence for clinical efficiency and cost‐effectiveness in the nonischemic etiology of heart failure, as most of the evidence of efficacy comes from patients with ischemic etiology. Aggressive campaigns aimed at increasing implementation of GDMT components, however have notably increased implantations of Defibrillators by 30.9%, the IMPROVE‐HF Registry reported.^5^

Universal compliance with this recommendation has subjected many patients to potentially arrhythmogenic and painful shocks, psychological trauma, worsened heart failure, and increased hospitalization, without any meaningful prolongation of their lives.

Conflicting results of the randomized trials that are cited in support, make the recommendation of ICD for HF patients debatable. The DANISH Trial did not show mortality benefit from defibrillators in nonischemic systolic heart failure^6^ and they were ineffective for patients in NYHA functional class III in the SCD‐HeFT trial.^7^ In contrast, the DEFINITE trial^8^ showed that ICDs are effective for NYHA III patients but not for patients in the NYHA II functional class. Overall there was merely a trend toward lower mortality in the DEFINITE trial cohort, mostly for those with nonischemic cardiomyopathy.

ESC 2016^2^ guideline states that “in patients with moderate or severe HF a reduction in sudden death may be partially or wholly offset by an increase in death due to worsening HF.”

Even in patients with ischemic cardiomyopathy and myocardial scarring, prophylactic implantation of defibrillators was not beneficial in the absence of spontaneous or inducible ventricular arrhythmia, as noted in the CABG‐PATCH trial and MADIT II cohort.^9^, ^10^, ^11^

A report of the mortality risk prediction model for HF patients with ICDs showed that patients with clinical features identified as “SHOCKED predictors” may have a 2 years mortality of almost 40% and may not benefit from ICD implantation for primary prevention. The predictors included the NYHA III functional class as well as six other features.^12^

Still, defibrillator implantation for primary prevention of sudden death is a class I recommendation^1^, ^2^ for patients with EF 35% or below who are expected to live for at least a year, irrespective of their NYHA functional class, stage of HF, and comorbid conditions. The recommendation does come with an advisory for “a discussion about the potential for sudden death and nonsudden death from HF or noncardiac conditions” with patients and their families. This advisory appears perfunctory, given the complexity of mortality, and the benefit predictions of ICDs for those patients. Furthermore, ICDs carry a CLASS IIb recommendation in the guidelines (“may be considered” by ESC “may be reasonable” by ACC/AHA, can be useful/effective) for these patients.

Risk of inappropriate ICD therapy (antitachycardia pacing plus shocks), ranging from 10% to 24% over 20‐45 months of follow up in major randomized trials of primary prevention of sudden death,^13^ compromises the quality of life of patients and is potentially arrhythmogenic. ICD shocks may cause increased hospitalization and worsening of heart failure, as reported in 19.9% of patients in the MADIT II trial.^14^

A more cost‐effective strategy than the present recommendation would be to stratify risk for sudden death in HF based on the inclusion criteria of the MADIT I trial^15^ or to restrict ICDs to patients, who have had either spontaneous or inducible ventricular arrhythmia. ICD did not reduce mortality in patients without inducible arrhythmia in the EP study in the MADIT II trial (although the EP study was not required for inclusion in the trial) (Mortality 16.6% in noninducible VT patients of treatment arm vs 19.8% in control).^11^

## 2 IVABRADINE AND HEART RATE REDUCTION IN HF, WHY NOT DIGOXIN?

McAlister et al,^16^ based on their analysis of landmark beta‐blocker trials, reported an 18% reduction in the risk of death with every 5‐bpm reduction in heart rate for HF patients. Ivabradine was included in the GDMT(class IIa) based on the SHIFT Trial^17^ in which despite a 9‐bpm reduction in heart rate, no mortality benefit was shown, perhaps because of the underutilization of beta‐blockers. Only 26% of the patient in the SHIFT trial were on a target dose of beta blockers,11% were not on any beta blockers and 15% were on nonguideline recommended beta blockers. The 26% reduction in heart failure hospitalization in the trial was much lower, than the landmark trials of beta‐blockers, which showed approximately similar posttreatment heart rates, but a 30%‐35% reduction in mortality and hospitalization for heart failure (Table 1). Not surprisingly, a recent report found a limited role for ivabradine in HF patients when beta‐blocker therapy was adequately optimized.^27^

**Table 1:** Beta blockers, ivabradine, and digoxin trials showing heart rate reduction and mortality and hospitalization outcomes

Trials	Heart rates bpm (mean)			All‐cause mortality %			Hospitalization %		
	Baseline	Mean reduction	Final	Treatment	Placebo	Reduction	Treatment	Placebo	Reduction
MDC^18^	83	15	75‐77	23	21	(NS)	20	28	8
MERIT‐HF^19^	83	16	65‐68	7.2	11	(32)	31	45	32
CIBIS^20^	83	15.7 +‐ 1.7	67	16.6	20.9	(NS)	24	34.5	NR
CIBIS II^21^	80	9.8	70	11.8	17.3	(34)	33	39	20
US‐HF^22^	84	12.6	NR	3.2	7.8	(65)	14.1	19.6	27
COPERNICUS^23^	83	12.5	71	11.4	18.5	(35)	17	23	24
ANZ^24^	76	9.5	66	9.6	12.5	(NS)	48	58	23
COMET^25^	81	12‐13	68‐69	8.3 (carvedilol)	10.0 (metoprolol)		36 (carvedilol)	36 (metoprolol)	
SHIFT^17^	80	9^#^	67	16	17	(NS)	16	21	26
DIG^26^	79	NR	NR	34.8	35.1	(NS)	26.8	34.07	28

COMET* (carvedilol V/S Metoprolol), SHIFT# Heart rate reduction relative to Placebo, absolute reduction 11 bpm. NR = Not Reported.

In patients truly intolerant to beta‐blockers due to hypotension or myocardial depression, reasons for digoxin (class IIb) not favored despite treatment experience of more than century, instead of ivabradine are not clearly explained in the guidelines. Digoxin, by increasing vagal tone while decreasing sympathetic tone, decreases heart rate and acts as a positive inotrope, and it is either neutral to blood pressure or increases it. Unlike ivabradine, digoxin decreases heart rate even in the presence of atrial fibrillation, which was reported to be present in 14%‐16% of patients with HF in the IMPROVE‐HF registry.^5^

In the DIG trial^26^ digoxin reduced the relative risk of the composite endpoint of death and hospitalization comparably to ivabradine (Table 1), even without concomitant beta‐blocker therapy.

The guidelines have relegated digoxin to only select patients with Stage C HF and persistent symptoms during GDMT because “long term trial with NYHA II or III HF treatment with digoxin had no effect on mortality but modestly reduced the combined risk of death and hospitalization” in the DIG trial.^1^, ^2^

The benefit analysis of the DIG trial^26^ may have been confounded by the fact that more than 75% of the enrolled patients were in NYHA I or II, with lower expected mortality and hospitalization. Additionally, 11.8% had supratherapeutic digoxin levels, shown to be associated with increased mortality. There was also a 37% higher hospitalization rate for unstable angina because of the high prevalence (60%‐65%) of myocardial infarction and coronary artery disease among the enrolled cohort.

In all landmark beta‐blocker trials showing a mortality benefit, 53%‐91% of randomized patients were also taking digoxin. Digoxin use was particularly prevalent in carvedilol trials, which showed the largest mortality benefits. This raises the questions of whether digoxin, with its modest ionotropic action, balances the myocardial depressant action of beta‐blockers and provides additional benefits particularly in patients with more advanced heart failure.

This hypothesis was examined in a retrospective analysis of four US Carvedilol‐HF trials^22^ and ANZ trial cohorts.^24^ The result of the analysis is summarized in Table 2.^28^ Although the authors reported, statistically inconclusive results, due to heterogeneity of the included data(ANZ trial data), clinical benefits of concomitant Digoxin therapy is obvious.

**Table 2:** 28 Comparison of combination of carvedilol ± digoxin effect with carvedilol or digoxin alone on all‐cause mortality and hospitalization in CHF

Carvedilol	
All cause hospitalization;	RESULT
Combination with digoxin:	32% Risk reduction
Alone without digoxin:	22% Risk reduction
All cause death and all cause hospitalization:	
Combination with digoxin:	37% Risk reduction
Alone without digoxin:	20% Risk reduction
Digoxin	
All cause hospitalization:	
Combination with carvedilol:	38% Risk reduction
Alone + placebo without carvedilol:	28% Risk reduction
All cause death and all cause hospitalization:	
Combination with carvedilol:	36% Risk reduction
Alone + placebo without carvedilol:	18% Risk reduction

Additive beneficial effect of digoxin over carvedilol has been shown in patients with heart failure and atrial fibrillation and is not limited to heart rate reduction alone.^29^

The apprehension of increased mortality with digoxin use, which has derived from observational reports utilizing statistical tools like “propensity matching” appears to exemplify what John Ferrier wrote while commenting on the “Purple Foxglove”: “The mischief of precipitate conclusions is nowhere more sensibly felt than in medical practice.”^30^ These reports contradict results of the only randomized trial of digoxin—the DIG trial, which showed a neutral effect of digoxin on mortality. The apparently increased mortality with digoxin is an example of “confounding by indication,” and is well‐exemplified by the results of landmark BHAT trial,^31^ where an initial odds ratio 2.87 of mortality associated with digoxin decreased to 1.07 after adjustment for 17 independent variables besides HF and complex ventricular premature beats, that were predictive of mortality.

## 3 ARNI (SECUBITRIL/VALSARTAN) UNSETTLED SAFETY AND COST‐EFFECTIVENESS

The 2017 update of the ACC guidelines^32^ recommends replacing ACE‐I or ARB (class IA) with ARNI (sacubitril/valsartan) (class I B‐R) for HFrEF NYHA II or III patients based on the PARADIGM‐HF^33^ results. However ESC^2^ recommends replacement only with persistent symptom despite optimal treatment with ACE‐I, beta Blockers, and MRAs. Hypotension and renal failure have been the major issues with ARNI use in real‐world practice similar to PARADIGM‐HF, in which 14.6% of patients failed the “run‐in‐phase.” During the trial, 16.7% of patient had symptomatic hypotension with 2.7% incidence of SBP of less than 90 mm Hg, incidence was even more (18%), in older patients, over 75 years. In the recently reported PIONEER‐HF trial^34^ with ARNI, symptomatic hypotension was the cause of discontinuation in 15% of the overall 20% discontinuation rate in addition to a 12% RUN‐IN phase withdrawal.

Attempted use of ARNI invariably forces clinicians to either discontinue or decrease the dose of beta‐blockers to avoid hypotension, which may have been the reason for almost 50% of the PARADIGM‐HF cohort reportedly receiving less than 50% of the target dose of beta‐blockers recommended in the guidelines. No attempt at optimization of beta‐blocker dose during the trial has been recorded and prioritization of Neprilysin inhibition over beta‐blockade for HF treatment has not been suggested by the trial.

There have also been hints of interactions between ARNI and beta‐blockers in the trial. The hazard ratio for the primary endpoint (composite of death from cardiovascular causes and first hospitalization with heart failure) was 0.79 in the subgroup of patients on less than 50% of the target dose of beta‐blockers. In comparison, the hazard ratio for patients on 50% or more of the target dose of beta‐blockers was 0.85. This was not statistically significant but raised concerns over combined treatment with beta‐blockers and ARNI in HF patients.

The long‐term safety of neprilysin inhibition also remains unsettled. Its association with Alzheimer's disease and the worsening of prostate and breast cancers is still being investigated. Bradykinin and Substance P buildup in patients on ACE‐I/ARB for more than 5 years have recently been reported to be associated with lung cancer.^35^ With an equal or greater role for neprilysin in bradykinin metabolism than ACE,^36^, ^37^ a faster substance P buildup in the lungs appears likely with a sacubitril/valsartan combination, since sacubitril also inhibits ACE.^38^ The potential for its association with lung cancer appears real, particularly in smokers and patients with COPD, which is present in 16.5%‐22.2% of HF patients.^5^

## 4 BETA BLOCKERS AND SPLIT THERAPEUTIC EFFECT IN ADVANCED HF

Despite unequivocal evidence associating maladaptive adrenergic activity with progression and pathogenesis of heart failure, beta blockade, and lowering norepinephrine levels have not been shown to be universally beneficial. The MOXCON(Central Sympatholytic) trial^39^ was terminated prematurely due to excessive mortality despite a 23% reduction in norepinephrine level in patients, 58% of whom were in NYHA class III and IV. Likewise, in BEST trial,^40^ in which 100% patients were in NYHA III and IV, a 19% reduction of norepinephrine with Bucindolol did not result in any mortality benefit, unlike other trials of beta‐blockers with more NYHA I, II, and III patients.

Landmark beta‐blocker trials mostly randomized patients in NYHA II and III, with less than 5% of patients belonging to NYHA functional class IV. Those studies cannot exclude a lack of benefit or even harm in patients with more advanced symptoms and a interaction of NYHA class and beta‐blocker therapy has never been adequately reported.

The differential effects of adrenergic blockade have been shown previously in patients with different severities of heart failure. While patients with NYHA II tolerated a complete sympathetic blockade with guanethidine, patients in the NYHA III and IV functional classes had decompensation and clinical worsening.^41^

Evidently intolerability of negative ionotropic effect of the beta blockade mostly led to exclusion of otherwise eligible HF patients with higher NYHA functional class in most of the landmark beta blocker trials. For example most of the Carvedilol trial and MDC trial^18^ with metoprolol had a predesigned “run‐in‐period.” During this phase, 4%‐6% (mean 5.3%) of eligible patients were excluded due to medication intolerance, clinical deterioration, or death. Analysis of the data shows that it was mostly patients in the higher NYHA functional class compared to the randomized cohort who were excluded in the RUN‐IN Phase. While Krum et al^42^ reported a 7% RUN‐IN Phase death in the cohort, 73% of whom were in NYHA III or IV, the PRECISE trial^43^ with 96% NYHA II or III patients reported 1.7% RUN‐IN Phase death.

A recent retrospective analysis from an outpatient clinical practice looking at the interaction between beta‐blockers and NYHA classes showed 3%, 9%, 13%, and 22% intolerability for NYHA classes I, II, III, and IV, respectively.^44^ An Australian transplant center reported worsening heart failure or mortality in 29% of NYHA IV patients treated with carvedilol as opposed to 19% in other NYHA functional classes (I/II/III).^45^ However, these results were not considered in periodic updates of the guidelines.

Guidelines recommend making “every effort to achieve target dose of beta‐blockers shown to be effective in major clinical trials.”^1^ This invariably leads to hypotension, fluid retention, and worsening HF, particularly in patients with NYHA III or IV symptoms, forcing increase in the dosage and numbers of diuretics, which has been shown to be a marker of increased mortality over 6 months in patients hospitalized with advanced heart failure in the ESCAPE trial.^46^

Increasing dosage of beta blockers and ACE‐I which cause hypotension (described as less than 80 mm Hg in the guideline^1^) and negative inotropy has a complex effect on renal function and overall sodium excretion, varying from mere alteration in diurnal excretion pattern to gross sodium retention, particularly in patients with NYHA IV CHF.^47^ Sodium retention becomes more pronounced when SBP runs low. Hypotension and decreased renal perfusion reverse “pressure natriuresis” in the absence of angiotensin II and norepinephrine‐induced renal vasoconstriction, as shown with guanethidine administration in normal humans.^48^, ^49^ Angiotensin II dependence for maintaining GFR in severe heart failure is heightened by the common occurrence of excessive diuresis—the latter leading to worsened renal function with sodium depletion and dehydration.

This pathophysiology of advanced heart failure may have been the cause of 44% of the deaths in NYHA IV patients within 3 months of metoprolol initiation reported by Waagstein et al.^50^ On the other hand, NYHA III patients without any right heart dysfunction and less than 1 grade MR showed significant improvement with metoprolol. Withdrawal of metoprolol resulted in the deaths of 17% of NYHA class III patients, with 75% of deaths being sudden. Others showed worsened hemodynamics, except for the surviving NYHA IV patients, who did not show any deterioration. Re‐administration of metoprolol resulted in clinical improvement of all those in NYHA II or III, but 40% of patients with NYHA III symptoms required additional treatment with digoxin.

## 5 BETA BLOCKERS IN HEART FAILURE PATIENTS WITH OTHER CO‐MORBIDITIES

Adverse effects of beta blockers have also been reported in patients of heart failure with certain co‐morbid conditions. Bisoprolol has been reported to reduce cardiac output and 6 minute walk distance in patients with right ventricular dysfunction due to pulmonary hypertension,^51^ while Metoprolol succinate decreased cardiac index and increased NT‐pro BNP in patients with mild to moderate aortic stenosis even when LVEF was normal.^52^ Effectiveness of beta blockers in patients of heart failure with these common co‐morbidities have not been discussed in the guidelines, and their use appears mere extrapolation of the data of the trials. The observational report of heart failure hospitalization in the ARIC cohort showed that, initiation of GDMT reduced mortality at 1 year, but 23% patients needed GDMT modification predicted by selected comorbidities and disease acuity.^53^

These reports may suggest that patients with advanced heart failure are better served with withdrawal or reduction in dosage of beta‐blockers. NYHA III, C patients may need digoxin to balance overall myocardial depressant effect of beta‐blockers to reduce heart failure admissions and delay progression to Stage D.

Patients eligible for this strategy have been identified in routine practice by their repeated hospital admissions, escalated need for diuresis, low SBP, worsening renal function, and restrictive mitral inflow pattern with RV dysfunction on echocardiography despite continued GDMT.

## 6 EFFECT OF GDMT ON MORBIDITY COMPRESSION IN HEART FAILURE

Although GDMT have decreased mortality and impacted progression in patient with mild to moderate heart failure, realization of morbidity compression, and quality of life improvement for patients with more advanced HF has not been achieved fully. This appears to be suggested by the report of the temporal trends in the treatment and outcome of advanced HFrEF between 1993 and 2010. The study was divided into 6‐year eras. Although there was a 65% decrease in all‐cause mortality over the eras, there was an increase in protracted deaths due to “pump failure” from 13% in Era I to 21% in Era 3. Patients in Era 3 (2005‐2010) had higher PA pressure, PCWP, and systemic vascular resistance, but lower cardiac output and LVEF. Patients with worsening heart failure needed more urgent heart transplant and ventricular assist device placements to sustain life, than similar patients of Era 1 and Era 2.^54^

This is further supported by the seventh annual INTERMACS report of 2015. The report showed an increase in LVAD implantation as destination therapy from 14.7% in 2006 to 45.7% in 2014,^55^ either due to a sicker CHF population, wider availability of LVAD, or both. The AHA report of 2018 indicates HF as an underlying cause of death in death certificates and shows a 27.7% increase from 2005. The 30‐day readmission rate was 21.9% (20.2%‐24.1%), showing minimal change despite increasing use of GDMT.^56^

A recent observation report of optimization of GDMT following hospitalization showed, reduced 1 year mortality but no reduction of hospital readmission for heart failure.^57^

Generalization of treatment of heart failure to “Get With The Guideline” dissuades clinicians from individualizing treatment particularly for patients not represented adequately in heart failure trials such as patients older than 80 years, advanced Hf, African Americans and patients with right heart failure, pulmonary hypertension and significant valvular diseases.

Currently heart failure is the most expensive diagnostic‐related group for Medicare, because cost‐effectiveness of many of the recommendation for treatment is at best controversial. For example cost effectiveness ratio of ICD for primary prevention was below $ 100 000 only when effectiveness of ICD continued for at least 7 years^58^ and replacing ACE‐I by ARNI appears to be resulting in incremental cos‐effectiveness ratio of $ 143 891/QALY gained.^59^

A reappraisal of CHF guidelines and answers for some outstanding questions associated with GDMT are urgently needed. Till then clinicians should have flexibility of adjusting therapies based on differing clinical pictures and status of patients.

## CONFLICT OF INTEREST

The authors declare no potential conflict of interest.
